# The interaction between gut microbiota and host DNA methylation in the pathogenesis and therapy of inflammatory bowel disease

**DOI:** 10.3389/fmicb.2025.1718358

**Published:** 2025-12-04

**Authors:** Qing Jin, Xiang Li, Zhonggui Liu, Tingting Qi, Dewang Xiao, Wentai Yang

**Affiliations:** 1 Laboratory Medicine, The First Affiliated Hospital of Gannan Medical University, Ganzhou, Jiangxi, China; 2 First Clinical Medical College, Gannan Medical University, Ganzhou, Jiangxi, China; 3 Department of Gastroenterology, The First Affiliated Hospital of Gannan Medical University, Ganzhou, Jiangxi, China

**Keywords:** gut microbiota, DNA methylation, inflammatory bowel disease, epigenetics, multi-omics, therapeutic intervention

## Abstract

Inflammatory bowel disease (IBD) represents a multifaceted, chronic inflammatory disorder of the gastrointestinal tract, with its pathogenesis modulated by a confluence of genetic, environmental, and immunological factors. Recent advancements have underscored the significance of the interaction network between gut microbiota and host epigenetic regulation, particularly via DNA methylation, as a pivotal area of investigation in elucidating the mechanisms underlying IBD. The gut microbiota exerts influence on host gene expression through its metabolic byproducts, thereby modulating immune responses and intestinal barrier integrity, while changes in host DNA methylation status both reflect and mediate this complex interaction. This review delineates the molecular mechanisms that regulate the interplay between gut microbiota and host DNA methylation, examines the impact of environmental factors on the onset and progression of IBD through microbiota-epigenetic pathways, and highlights recent multi-omics research findings and their potential applications in precision medicine. The objective is to furnish a theoretical framework and research trajectory for deciphering the molecular underpinnings of IBD and fostering the development of innovative therapeutic strategies.

## 1 Introduction

Inflammatory bowel disease (IBD), which includes disorders such as Crohn’s disease and ulcerative colitis, has become a major global health issue, with its prevalence and incidence increasing markedly, especially in Western and newly industrialized nations. The etiology of IBD is multifactorial, involving a complex interaction among genetic predispositions, environmental factors, and microbial influences, which collectively contribute to the dysregulated immune response observed in individuals with the condition. Recent studies have highlighted that the composition and functionality of the gut microbiota play a pivotal role in the pathogenesis of IBD, acting as both a modulator of immune responses and a contributor to the inflammatory processes that characterize these diseases (Ceballos et al., 2021). The rising incidence of IBD has been paralleled by shifts in dietary patterns and lifestyle, which are believed to influence gut microbiota dynamics, thereby exacerbating the inflammatory state (Vieujean et al., 2022).

A crucial factor in the development of IBD is the impact of epigenetic changes, especially DNA methylation, which can modify gene expression without altering the DNA sequence itself (Corridoni et al., 2020). This epigenetic regulation plays a pivotal role in mediating the influence of environmental factors, such as dietary habits and microbial composition, on immune responses and their contribution to inflammation (Vieujean et al., 2022). Aberrant DNA methylation patterns have been associated with the pathogenesis of IBD, indicating that epigenetic mechanisms may act as an intermediary connecting environmental factors to the manifestation of the disease (Mu et al., 2023). Moreover, the interplay between gut microbiota and host epigenetics offers a promising avenue for elucidating the complexities of IBD pathogenesis. Alterations in microbial communities can induce significant modifications in the host’s epigenetic landscape, thereby impacting immune function and inflammatory responses (Kim et al., 2024).

The complex interplay between gut microbiota and epigenetic regulation is of significant academic interest, highlighting the potential for therapeutic interventions aimed at these pathways. The advancement of multi-omics methodologies, which integrate genomic, transcriptomic, metabolomic, and epigenomic data, has yielded profound insights into the mechanisms underlying IBD and has facilitated the exploration of novel personalized treatment strategies (Mu et al., 2023). Through the elucidation of molecular interactions between microbial communities and host epigenetics, researchers are identifying novel biomarkers and therapeutic targets, which hold the potential to enhance treatment efficacy and improve patient outcomes (Zhang et al., 2025).

In recent years, advancements in high-throughput sequencing technologies, coupled with the application of multi-omics analyses, have significantly transformed our understanding of IBD. These methodologies enable a comprehensive investigation of the interactions among host genetics, microbial communities, and environmental factors, thereby facilitating the identification of disease-specific biomarkers and the development of targeted therapeutic interventions (Mu et al., 2023). As the field advances, there is an increasing focus on the necessity for personalized medicine strategies that account for individual differences in microbiota composition and epigenetic profiles, thus customizing interventions to address the specific needs of each patient (Mu et al., 2023). The transition towards precision medicine signifies a substantial advancement in the management of IBD, offering the potential to improve treatment efficacy and reduce the adverse effects commonly associated with traditional therapeutic approaches.

In summary, the growing understanding of the interactions between the gut microbiota and host DNA methylation is reshaping our approach to studying IBD. As research continues to unravel the complexity of these interactions, there is hope for developing innovative therapeutic strategies that leverage these insights to improve patient care and treatment outcomes for IBD. This review clarifies the molecular mechanisms regulating the interaction between gut microbiota and host DNA methylation and introduces corresponding intervention strategies. It aims to provide a theoretical framework and research pathway for deciphering the molecular mechanisms of IBD, thereby advancing the development of innovative therapeutic strategies.

## 2 The role of gut microbiota in the pathogenesis of IBD

### 2.1 Gut microbial diversity and dysbiosis

The gut microbiota plays an essential role in maintaining host health, particularly in the context of IBD. Recent research has demonstrated that individuals with IBD exhibit a marked reduction in gut microbial diversity, characterized by a decrease in beneficial probiotics and an increase in pathogenic bacteria (Ji et al., 2023). This dysbiosis is considered a critical factor in the pathogenesis of IBD, as it can lead to aberrant activation of the immune system, thereby promoting intestinal inflammation (Santonocito et al., 2024). For example, the depletion of beneficial microbial populations, such as those producing short-chain fatty acids (SCFAs), can compromise intestinal barrier function and amplify the inflammatory response (Wang et al., 2024). In patients with IBD, there is frequently a significant reduction in SCFA-producing bacteria, which are vital for maintaining gut homeostasis and modulating immune responses (Bi et al., 2025). The imbalance in gut microbiota composition not only exacerbates inflammation but may also contribute to the persistence of the disease by perpetuating a cycle of immune dysregulation and microbial imbalance. Moreover, pathogenic bacteria, including adherent-invasive *Escherichia coli*, have been observed to be more prevalent in the gastrointestinal tracts of patients with IBD, thereby adding complexity to the disease’s pathophysiology (Shoham et al., 2025). These observations emphasize the critical role of gut microbiota diversity in IBD and suggest potential therapeutic strategies aimed at restoring microbial equilibrium to alleviate disease severity. Interventions such as probiotics, dietary modifications, and fecal microbiota transplantation are currently under investigation as methods to enhance gut microbial diversity and improve clinical outcomes for IBD patients (Li et al., 2024). The complex interplay between gut dysbiosis and immune activation represents a promising area for future research, with the potential to identify novel biomarkers for disease progression and treatment response in IBD. Understanding the influence of specific microbial populations on immune function and inflammation could lead to the development of targeted therapies that address not only the symptoms of IBD but also the underlying dysbiosis contributing to the disease’s pathology (Sultan et al., 2021; Takahashi et al., 2024).

### 2.2 Immune regulatory functions of microbial metabolites

Microbial metabolites are integral to the modulation of immune responses, exerting a substantial impact on the functionality of various immune cells. Notably, SCFAs and tryptophan metabolites are distinguished by their diverse regulatory effects on immune cell functions. SCFAs, including acetate, propionate, and butyrate, are generated through the fermentation of dietary fibers by gut microbiota. These metabolites have been demonstrated to enhance the production of anti-inflammatory cytokines, facilitate the differentiation of regulatory T cells (Tregs), and fortify intestinal barrier function (Zhao et al., 2022). Consequently, they play a pivotal role in maintaining gut homeostasis and preventing inflammation. Furthermore, SCFAs have the capacity to activate G-protein coupled receptors (GPCRs) on immune cells, resulting in modified immune responses that may either facilitate tolerance or augment immune activation, contingent upon the specific context (Fu et al., 2023). Metabolites of tryptophan, especially those originating from the indole pathway, are crucial in the regulation of immune function. These metabolites have the capacity to activate the aryl hydrocarbon receptor (AhR), a key player in modulating immune responses and maintaining the integrity of the gut barrier (Pei et al., 2025). Through these mechanisms, microbial metabolites have the capacity to modulate the equilibrium between pro-inflammatory and anti-inflammatory responses, thereby influencing the pathogenesis of various diseases, such as IBD and colorectal cancer (Yin et al., 2024).

Furthermore, microbial metabolites influence the integrity of the intestinal barrier via receptor-mediated signaling pathways. Specifically, SCFAs have been demonstrated to upregulate the expression of tight junction proteins in intestinal epithelial cells, a critical process for preserving the barrier function of the gut epithelium (Yang et al., 2023). This is especially significant within the context of IBD, where heightened intestinal permeability may result in the translocation of luminal antigens and bacteria, subsequently provoking an inappropriate immune response (Iyer and Corr, 2021). Furthermore, tryptophan metabolites have the capacity to modulate the expression of genes linked to the epithelial barrier and immune tolerance, thereby underscoring the significance of microbial metabolites in maintaining gut health (Liu et al., 2025). The interaction between microbial metabolites and the immune system underscores the critical importance of sustaining a balanced gut microbiota. Dysbiosis can result in altered metabolite production, which may contribute to immune dysfunction and the progression of disease (Wang W. et al., 2025).

In conclusion, microbial metabolites, including SCFAs and tryptophan derivatives, serve as crucial regulators of immune cell function and the integrity of the intestinal barrier. These metabolites exert their effects through complex signaling pathways involving various receptors, thereby influencing the host’s immune responses. A comprehensive understanding of these interactions offers valuable insights into the mechanisms underlying immune-related diseases and underscores the potential for therapeutic interventions aimed at modulating microbial metabolism to restore immune equilibrium and enhance gut health. As ongoing research continues to elucidate the intricate relationships among gut microbiota, their metabolites, and the immune system, innovative strategies for managing inflammatory diseases may emerge, with a focus on dietary modulation and microbiota-targeted therapies (Niekamp and Kim, 2023).

### 2.3 Interaction between microbiota and gut barrier function

The gut microbiota is integral to preserving the integrity of the intestinal barrier, a critical function for preventing the translocation of deleterious substances and pathogens into systemic circulation. Dysbiosis has been demonstrated to compromise the epithelial barrier, resulting in heightened intestinal permeability, commonly referred to as “leaky gut.” This compromise permits the infiltration of inflammatory mediators and toxins through the intestinal lining, thereby initiating systemic inflammation and contributing to the pathogenesis of various gastrointestinal disorders, such as IBD (Zhou et al., 2024). For example, research has shown that changes in microbial composition can undermine the integrity of tight junctions, which are essential for preserving epithelial barrier function. Elevated levels of pathogenic bacteria, including specific strains of *Escherichia coli*, have been linked to the disruption of the gut barrier, leading to increased permeability and inflammation (Liu et al., 2023). Moreover, metabolites generated by the gut microbiota, such as SCFAs, have been associated with the fortification of the gut barrier through the upregulation of tight junction protein expression and the enhancement of mucosal immunity (Fei et al., 2024).

Conversely, impairment of the intestinal barrier may initiate a detrimental cycle that intensifies dysbiosis. Compromise of this barrier facilitates the translocation of bacteria and their byproducts, which subsequently disrupts the composition of the microbiota and sustains inflammatory processes (Yang et al., 2021). For instance, in conditions like ulcerative colitis, the inflammatory milieu may preferentially select for pathogenic microorganisms that flourish in a dysbiotic state, whereas commensal microbes that support barrier integrity are reduced (Fei et al., 2024). This interaction underscores the reciprocal relationship between the gut microbiota and the intestinal barrier. As the integrity of the barrier diminishes, dysbiosis becomes more pronounced, thereby initiating a cycle of inflammation and further compromising barrier function.

Furthermore, therapeutic strategies focused on reestablishing gut microbiota equilibrium, including the administration of probiotics, prebiotics, and dietary modifications, have demonstrated potential in improving gut barrier integrity and mitigating inflammatory responses (Chargo et al., 2024). For example, research has shown that probiotics enhance epithelial barrier integrity by modulating the composition of the gut microbiota, which in turn reduces intestinal permeability and systemic inflammation (Liu et al., 2025). Furthermore, dietary constituents, particularly fiber, can facilitate the proliferation of beneficial microbiota that synthesize SCFAs, which subsequently contribute to the preservation of an intact and functional gut barrier (Li et al., 2023).

In summary, the relationship between gut microbiota and intestinal barrier function is intricate and multifaceted. Dysbiosis can result in barrier dysfunction, while compromised barrier integrity can exacerbate microbial imbalances, perpetuating a harmful cycle that contributes to the pathogenesis of various gastrointestinal diseases. Comprehending these interactions is essential for the development of targeted therapeutic strategies aimed at restoring gut health and preventing disease progression.

## 3 Regulatory role of host DNA methylation in IBD

### 3.1 Basics of DNA methylation and its function in gene expression

DNA methylation represents a pivotal epigenetic modification integral to the regulation of gene expression and the maintenance of genomic stability. The most extensively characterized form of DNA methylation occurs at the 5′ position of cytosine residues, particularly within CpG dinucleotides, leading to the formation of 5-methylcytosine (5mC). This modification predominantly localizes to the promoter regions of genes, where it exerts an influence on transcriptional activity. Typically, methylation within these regions functions as a repressive signal, obstructing the binding of transcription factors and RNA polymerase, thereby inhibiting gene expression. Empirical studies have illustrated that aberrant DNA methylation patterns are implicated in the silencing of tumor suppressor genes across various cancers. For example, in colorectal cancer, hypermethylation of promoter regions is correlated with diminished gene expression and adverse clinical outcomes (Hong and Rhee, 2022). Moreover, the dynamic characteristics of DNA methylation facilitate the modulation of gene expression in response to environmental stimuli, including variations in nutrient availability and stress conditions. This adaptability is essential for cellular differentiation and the immune response, as it permits cells to precisely adjust their gene expression profiles in accordance with their functional demands. For instance, alterations in DNA methylation have been associated with the differentiation of immune cells, wherein specific genes are either activated or repressed to elicit an appropriate immune response (Otsuki et al., 2022). Therefore, comprehending the mechanisms underlying DNA methylation and its influence on gene expression is crucial for elucidating the pathogenesis of various diseases and for the development of targeted therapeutic strategies.

DNA methylation plays a multifaceted role that extends beyond simple transcriptional repression, encompassing the regulation of immune cell differentiation and the modulation of inflammatory responses. Empirical studies have demonstrated that the dynamics of DNA methylation are critical during the development of immune cells, as they influence the expression of genes pivotal to immune function. For example, in T lymphocytes, the demethylation of particular genes is imperative for their activation and subsequent differentiation into effector cells, which are vital for mounting an effective immune response (Vural et al., 2021). In contrast, hypermethylation may result in the silencing of genes that typically promote inflammation, thus facilitating immune tolerance and maintaining homeostasis. This delicate equilibrium is crucial for the prevention of autoimmune diseases, as disruptions in DNA methylation patterns can lead to improper immune activation and subsequent tissue damage (Rauluseviciute et al., 2020). Furthermore, the interaction between DNA methylation and other epigenetic modifications, such as histone modifications, adds complexity to the regulatory framework of gene expression. For example, the binding of methyl-binding domain proteins to methylated DNA can result in the establishment of repressive chromatin configurations, thereby effectively silencing gene expression (Chen et al., 2021). This complex regulation highlights the critical role of DNA methylation in modulating not only the expression of individual genes but also in coordinating extensive transcriptional programs vital for cellular identity and function. The investigation of DNA methylation and its impact on gene expression represents a rapidly advancing field with profound implications for elucidating disease mechanisms and developing innovative therapeutic strategies.

### 3.2 Aberrant methylation profiles of genes associated with IBD

Recent research has increasingly underscored the importance of aberrant DNA methylation patterns in IBD, with a particular focus on inflammation-related genes such as TNF-*α* and IL-6. These genes serve as pivotal mediators in the inflammatory response, and their expression is meticulously regulated by epigenetic mechanisms, including DNA methylation. A study demonstrated that the methylation of the TNF promoter is significantly diminished in inflamed mucosa compared to uninflamed segments in patients with IBD, correlating with the severity of the disease (Levic et al., 2024). The observed hypomethylation is posited to augment TNF-α expression, thereby sustaining the inflammatory cycle that is emblematic of IBD. In a similar vein, IL-6, a critical cytokine within the inflammatory cascade, has demonstrated altered methylation patterns in IBD, which further contribute to the dysregulation of immune responses (Akanyibah et al., 2024). The aberrant methylation of these genes induces a dysregulation in their expression, culminating in a persistent chronic inflammatory condition that proves challenging to ameliorate.

Moreover, the association between aberrant methylation patterns and IBD encompasses not only specific genes but also more extensive epigenetic landscapes. Genome-wide investigations have identified a multitude of differentially methylated regions (DMRs) linked to IBD, highlighting the intricate nature of epigenetic regulation within this framework. For example, a meta-analysis demonstrated that numerous DMRs are enriched in genes related to immune response and inflammation, indicating that the dysregulation of these pathways may represent a prevalent characteristic among IBD patients (Joustra et al., 2023). This dysregulation is not simply a consequence of the disease; rather, it may contribute causally to its pathogenesis. Aberrant methylation patterns have the potential to alter gene expression profiles, thereby promoting inflammatory processes.

The sustained presence of aberrant methylation patterns may have significant implications for therapeutic responses in IBD. For instance, individuals exhibiting particular methylation profiles in genes such as NLRP3 have demonstrated differential responses to glucocorticoid therapy (Zudeh et al., 2025). This suggests that methylation status could potentially function as a predictive biomarker for treatment efficacy. This underscores the potential of employing DNA methylation patterns not only as biomarkers for disease status but also as targets for therapeutic intervention.

### 3.3 DNA methylation and dysfunction of intestinal epithelial cells

DNA methylation is pivotal in modulating the proliferation and differentiation of intestinal stem cells, which are vital for preserving the integrity and functionality of the intestinal epithelium. Methylation patterns are subject to dynamic alterations in response to a variety of physiological and pathological stimuli, thereby influencing the regenerative capacity of intestinal epithelial cells (IECs). Notably, the DNA methyltransferase DNMT3A has been identified as essential for maintaining the methylation landscape that regulates intestinal epithelial homeostasis. In studies utilizing murine models of ulcerative colitis (UC), it has been observed that the downregulation of DNMT3A results in global hypomethylation, leading to impaired regenerative capacity and reduced transepithelial resistance (Ansari et al., 2023). The observed hypomethylation was correlated with a decreased expression of tight junction proteins, which are essential for preserving epithelial barrier integrity. As a result, the diminished capacity of IECs to proliferate and differentiate efficiently undermines epithelial repair mechanisms, thereby exacerbating the pathogenesis of IBD, such as UC (Cheng et al., 2022). Moreover, aberrant methylation may induce apoptosis in epithelial cells, thereby further compromising barrier function and heightening vulnerability to inflammation and infection. For instance, in the context of HIV infection, altered DNA methylation patterns in colonic epithelial cells have been associated with increased epithelial barrier disruption and microbial translocation, underscoring the adverse impact of dysregulated methylation on intestinal integrity (Premadasa et al., 2025).

Moreover, aberrant methylation of particular genes has been implicated in the apoptosis of IECs. Alterations in methylation patterns can lead to the silencing of genes essential for cell survival and barrier integrity, resulting in heightened cell death and a compromised epithelial layer. Empirical studies have shown that hypermethylation of the tumor necrosis factor (TNF) promoter is correlated with mucosal inflammation in patients with IBD, indicating that methylation status may have a direct impact on disease severity and the viability of epithelial cells (Levic et al., 2024). The interaction between DNA methylation and the expression of genes associated with inflammatory responses highlights the critical role of epigenetic regulation in preserving intestinal epithelial homeostasis. Furthermore, research has demonstrated that the gut microbiota can modulate DNA methylation patterns, thereby adding complexity to the relationship between microbial exposure and epithelial health (Narabayashi et al., 2022).

## 4 Molecular mechanisms through which gut microbiota influence host DNA methylation

### 4.1 Microbial metabolite-mediated methylation regulation

**Figure 1:**
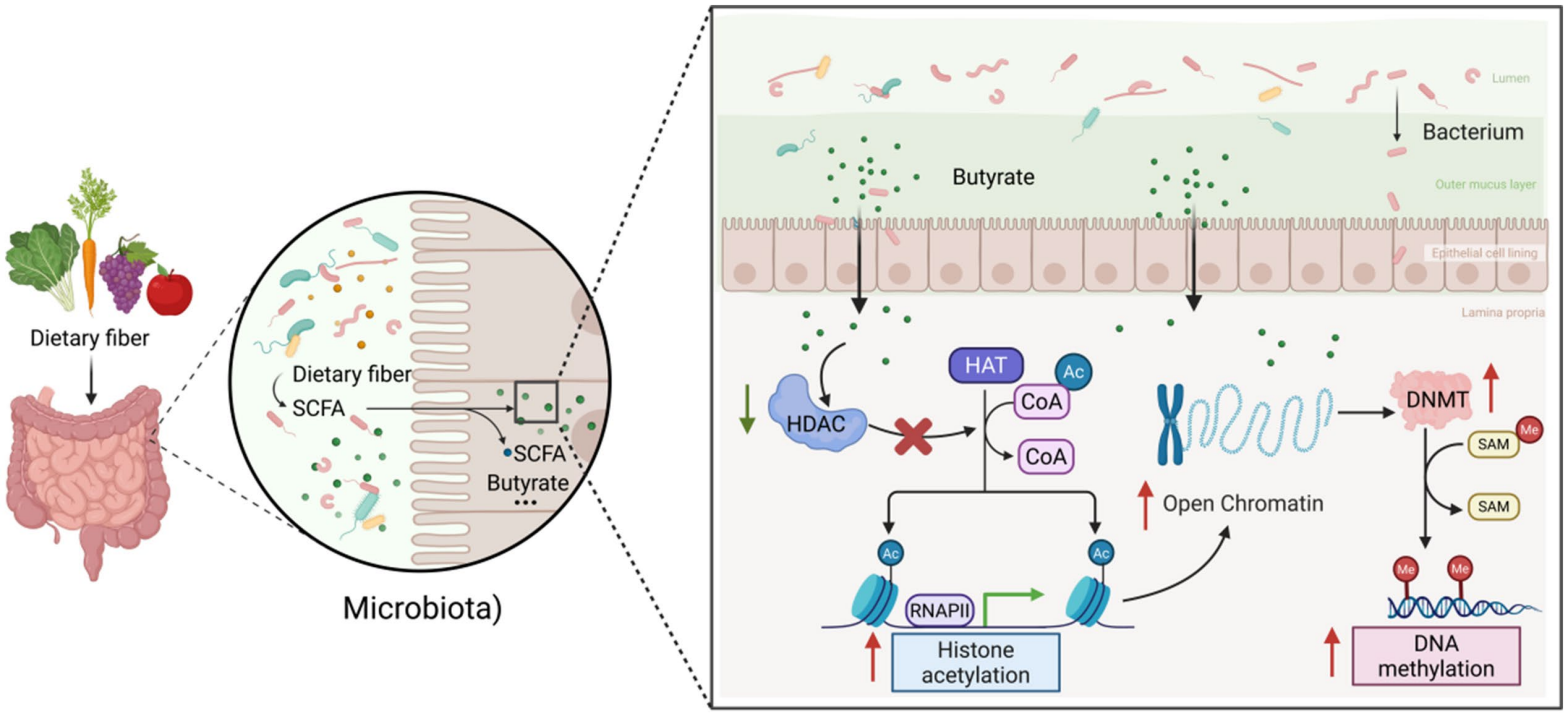
Schematic diagram of short-chain fatty acids (butyric acid) influencing DNA methylation by inhibiting HDAC and regulating DNMT: Dietary fiber undergoes fermentation by the gut microbiota, producing short-chain fatty acids represented by butyrate. As a key signaling molecule, butyrate reshapes chromatin structure and gene expression profiles at the epigenetic level by inhibiting histone deacetylases (HDACs) and indirectly affecting the activity of DNA methyltransferases (DNMTs).

Microbial metabolites, particularly SCFAs such as butyrate, are integral to the regulation of DNA methylation, primarily through their modulation of histone deacetylases (HDACs) and DNA methyltransferases (DNMTs) (Figure 1) (Paraskevaidis et al., 2025). Butyrate, a significant SCFA generated by the fermentation of dietary fibers by gut microbiota, functions as a potent inhibitor of HDACs (Zhang et al., 2025). This inhibition leads to increased histone acetylation, resulting in alterations in gene expression. This mechanism indirectly influences DNA methylation processes by affecting the availability and activity of methyl donors, such as S-adenosylmethionine (SAM), which are crucial for methylation reactions. The inhibition of HDACs by butyrate induces a more open chromatin conformation, thereby facilitating the access of DNMTs to DNA and potentially enhancing the methylation of specific genes involved in inflammation and immune responses. Moreover, the interaction between SCFAs and the host’s epigenetic mechanisms indicates that microbial metabolites have the capacity to modulate the epigenetic framework, thereby playing a role in the pathogenesis of IBD and other metabolic disorders. Empirical evidence demonstrates that dietary interventions designed to enhance the population of SCFA-producing bacteria can induce substantial alterations in the methylation patterns of genes linked to immune regulation and inflammation (Alsharairi, 2023; Guo et al., 2025). This underscores the therapeutic potential of dietary modulation as a strategy for managing IBD.

In addition to SCFAs, microbial-derived methyl donors, such as folate and vitamin B12, are integral to methylation reactions (Alharthi et al., 2022). Folate, a B-vitamin produced by specific gut bacteria, is essential for the synthesis of S-adenosylmethionine (SAM), the principal methyl donor in these processes (Alharthi et al., 2022). The availability of folate within the gut is contingent upon the composition of the gut microbiota, which exhibits considerable interindividual variability. Such variability can result in differences in the efficiency of methylation reactions, thereby influencing the host’s epigenetic regulation and disease susceptibility. For example, reduced folate levels have been correlated with increased alterations in DNA methylation, which may facilitate the development of colorectal cancer—a condition associated with dysbiosis and altered microbial metabolism (Su et al., 2016). Furthermore, vitamin B12, an essential methyl donor, is synthesized by certain gut bacteria and plays a crucial role in sustaining proper methylation patterns.

The interaction between microbial metabolism and host methylation processes highlights the significance of a balanced gut microbiome in preserving epigenetic health and preventing disease. Consequently, interventions aimed at enhancing the production of these microbial metabolites may offer promising strategies for modulating DNA methylation and improving health outcomes in individuals with IBD and other related conditions (Grycová et al., 2022).

### 4.2 Microbial-induced immune signals and epigenetic regulation

The interaction between microbially-induced immune signals and epigenetic regulation represents a rapidly expanding field of study that sheds light on the mechanisms by which gut microbiota can modulate host immune responses and gene expression through epigenetic modifications. The activation of inflammatory signaling pathways by microbial agents, particularly through the host immune system’s recognition of pathogen-associated molecular patterns (PAMPs), is pivotal in influencing the expression of DNMTs. These enzymes are responsible for the addition of methyl groups to DNA, thereby modulating gene expression without altering the DNA sequence itself. For example, microbial metabolites such as SCFAs can inhibit HDACs, resulting in increased histone acetylation and a more accessible chromatin structure, which facilitates the transcription of genes associated with immune responses (Reva et al., 2023). This process underscores the dynamic characteristics of the immune system, wherein the presence of distinct microbial communities can induce differential expression of DNMTs, consequently impacting the methylation patterns of genes implicated in inflammation and immune tolerance. Moreover, the inflammatory microenvironment resulting from dysbiosis can facilitate alterations in DNA methylation patterns, thereby establishing a feedback loop in which modified gene expression further affects microbial composition and immune responses (Mostafavi Abdolmaleky and Zhou, 2024). This complex relationship highlights the significance of comprehending the modulation of epigenetic mechanisms by microbial signals, potentially leading to the development of innovative therapeutic strategies that target microbiota-epigenetic pathways for the treatment of inflammatory diseases.

Beyond the activation of inflammatory pathways, the inflammatory microenvironment has the capacity to instigate dynamic alterations in methylation patterns. Persistent inflammation, frequently arising from dysbiosis, can trigger the activation of pro-inflammatory cytokines that modulate the expression of DNMTs and other epigenetic regulators. For instance, the presence of inflammatory cytokines, including tumor necrosis factor-alpha (TNF-*α*) and interleukin-6 (IL-6), can result in the upregulation of DNMTs, thereby promoting hypermethylation of genes essential for maintaining immune homeostasis (Xu et al., 2022). Epigenetic reprogramming may lead to a disruption of immune tolerance and heightened vulnerability to autoimmune diseases. Furthermore, the interaction between microbial metabolites and the host immune system can establish a feedback loop in which inflammation intensifies dysbiosis, perpetuating a cycle of immune dysregulation and epigenetic modifications. These insights into microbially-induced immune signals and their epigenetic ramifications not only deepen our comprehension of the pathogenesis of inflammatory bowel diseases but also underscore potential therapeutic strategies aimed at targeting these pathways to restore immune equilibrium and prevent disease progression (Zhang et al., 2025).

In summary, the interaction between microbial signals and epigenetic regulation is intricate and multifaceted, characterized by a dynamic interplay between the host immune system and the gut microbiota. The interactions between specific microbial taxa, their metabolites, and host DNA methylation patterns contribute significantly to IBD pathogenesis (Table 1). As ongoing research elucidates the mechanisms underpinning these interactions, it is becoming increasingly evident that targeting the microbiome and its epigenetic influences may offer a promising approach for the prevention and treatment of inflammatory diseases, such as IBD and other related conditions.

**Table 1:** Summary of microbial taxa, affected methylation targets, and associated IBD phenotypes.

Microbial taxonomic group/Factor	Affected methylation targets/pathways	IBD phenotype/Functional impact	References
SCFA-producing bacteria	-Genome-wide methylation landscape - Inflammation/immunity-related genes (indirectly affected via HDAC inhibition) - Treg cell differentiation-related genes (e.g., Foxp3)	- Enhanced intestinal barrier function - Anti-inflammatory environment - Immune tolerance - Reduced disease severity	Zhang et al. (2025), Alsharairi (2023), Guo et al. (2025), Wu et al. (2024), and Wang and Geng (2023)
Pathogenic bacteria	- Hypomethylation of pro-inflammatory cytokine genes (e.g., TNF-*α*, IL-6) - Abnormal methylation in immune response pathways	- Intestinal barrier disruption (“leaky gut”) - Pro-inflammatory state - Persistent chronic inflammation - Increased disease activity	Liu et al. (2023), Levic et al. (2024), Akanyibah et al. (2024), Meng et al. (2024), and Yan et al. (2023)
Tryptophan-metabolizing bacteria	- Methylation status of AhR target genes - Genes associated with epithelial barrier and immune tolerance	- Maintenance of intestinal barrier integrity - Immune regulation	Pei et al. (2025), Liu et al. (2025), and Grycová et al. (2022)
Environmental factors (high-fat/high-sugar diet, pollutants)	- Altered genome-wide methylation patterns - Abnormal methylation of genes in metabolic and inflammatory pathways	- Dysbiosis - Systemic Inflammation - Increased Susceptibility and Severity of IBD	Ji et al. (2023), Shi et al. (2024), Wang et al. (2025), Gill et al. (2022), and Chen et al. (2023)
Early-Life Microbial Exposure	Programmed methylation alterations in genes associated with immune system development	- Immune System Maturation - Long-Term IBD Risk Programming	Noble et al. (2023), Lepeule et al. (2024), Borowitz (2022), and Zachariah et al. (2024)

## 5 The impact of environmental factors on gut microbiome–host DNA methylation interactions

### 5.1 The impact of food additives and environmental pollutants

Food additives, notably titanium dioxide (TiO2), have been demonstrated to disrupt gut microbiota, subsequently influencing DNA methylation patterns. TiO2 is extensively utilized as a whitening agent in a variety of food products and has been classified as a potential carcinogen by the International Agency for Research on Cancer. The consumption of TiO2 nanoparticles can modify the composition of gut microbiota, resulting in dysbiosis, which is characterized by an imbalance in microbial populations. Empirical studies have indicated that exposure to TiO2 can exacerbate intestinal inflammation, thereby affecting the host’s epigenetic landscape. This dysbiosis may facilitate alterations in DNA methylation, a critical mechanism for regulating gene expression and maintaining cellular homeostasis. For example, changes in the gut microbiota resulting from TiO2 exposure can result in elevated levels of pro-inflammatory cytokines, which are linked to epigenetic modifications that enhance inflammatory pathways (Ji et al., 2023). These alterations may play a role in the development of IBD and other metabolic disorders, underscoring the necessity for further investigation into the long-term impacts of food additives on gut health and epigenetic regulation.

Environmental pollutants, encompassing heavy metals and microplastics, exert a substantial impact on oxidative stress and modify the host’s epigenetic landscape. Notably, cadmium (Cd) exposure has been linked to adverse alterations in gut microbiota composition and functionality. Elevated Cd levels can promote the proliferation of pathogenic bacteria while diminishing the populations of beneficial microbes. This microbial imbalance may exacerbate oxidative stress, which is recognized for inducing epigenetic modifications, including alterations in DNA methylation patterns. Furthermore, microplastics, which are increasingly pervasive in the environment, have been demonstrated to disrupt the equilibrium of gut microbiota and compromise the integrity of the intestinal barrier. Recent research suggests that microplastics have the potential to induce inflammation and oxidative stress, which may further contribute to epigenetic modifications that predispose individuals to a range of health conditions, including IBD (Shi et al., 2024; Wang Z. et al., 2025). The interplay between environmental pollutants and the gut microbiota highlights the critical need to understand how these factors collectively influence host health through epigenetic pathways. Therefore, addressing exposure to these pollutants is essential for mitigating their detrimental effects on human health.

### 5.2 The regulatory role of dietary structure and nutritional components

Diets rich in fats and sugars have been extensively associated with dysbiosis of the gut microbiota, potentially resulting in aberrant DNA methylation patterns. Such dietary habits disrupt the microbial ecosystem’s balance, promoting the growth of pathogenic bacteria and reducing the prevalence of beneficial strains. Specifically, the intake of excessive saturated fats and simple sugars has been demonstrated to modify the gut microbiota composition, resulting in an increase in pro-inflammatory bacteria and a decrease in short-chain fatty acid-producing species, which are essential for sustaining gut health and modulating immune responses (Gill et al., 2022). This dysbiosis not only intensifies inflammation but also affects epigenetic modifications, notably DNA methylation, which can modify gene expression associated with inflammatory and metabolic pathways. Studies suggest that metabolites produced by gut microbiota, such as short-chain fatty acids, are crucial in modulating gene expression via epigenetic mechanisms, including the regulation of DNA methylation (Chen et al., 2023). Consequently, the intake of diets rich in fats and sugars may significantly influence the composition of gut microbiota and the epigenetic regulation of the host, thereby playing a critical role in the pathogenesis of inflammatory bowel diseases (IBD) and various metabolic disorders.

In contrast, dietary fibers have been demonstrated to facilitate the proliferation of beneficial gut microbiota, which can exert a favorable impact on DNA methylation patterns. These fibers act as substrates for fermentation by gut microbiota, resulting in the production of short-chain fatty acids, such as butyrate, which have been linked to anti-inflammatory effects and enhanced gut barrier function (Wu et al., 2024). Short-chain fatty acids function as signaling molecules that modulate gene expression via epigenetic modifications. Specifically, they promote histone acetylation and inhibit histone deacetylases, thereby fostering a more conducive epigenetic environment (Wang and Geng, 2023). Moreover, the consumption of dietary fibers is associated with enhanced microbial diversity, a factor essential for sustaining gut health and preventing dysbiosis. Empirical studies have shown that elevated fiber intake is positively correlated with the increased prevalence of beneficial bacterial genera, such as Bifidobacterium and Lactobacillus, which are linked to improved metabolic health and decreased inflammation (Zheng et al., 2024). Consequently, dietary fibers facilitate the proliferation of beneficial gut microbiota and play a role in the regulation of DNA methylation and other epigenetic modifications. This underscores their potential as therapeutic agents in the prevention and management of inflammatory bowel diseases and associated metabolic disorders.

In conclusion, the interaction between dietary composition, specifically the equilibrium of macronutrients, and the gut microbiota plays a critical role in the regulation of DNA methylation and the modulation of disease outcomes. Diets high in fats and sugars are associated with dysbiosis and aberrant methylation patterns, whereas dietary fibers support a healthy microbiota and favorable epigenetic modifications. These findings highlight the significance of dietary interventions in influencing gut health and epigenetic regulation, presenting promising strategies for the prevention and treatment of inflammatory bowel diseases and various metabolic disorders.

### 5.3 The enduring impact of early-life environmental exposures

The perinatal period represents a crucial phase for environmental exposures that may exert enduring effects on immune system development and overall health. Empirical studies have demonstrated that exposure to microbial environments during gestation and early life stages can markedly alter DNA methylation patterns, subsequently influencing the maturation of the immune system. For example, maternal exposure to a diverse range of microbiota during pregnancy can induce epigenetic modifications in the offspring, thereby enhancing immune responsiveness and potentially mitigating the risk of IBD in later life (Noble et al., 2023). This epigenetic regulation is of paramount importance, as it influences the expression of genes integral to immune function and inflammatory responses. Moreover, detrimental environmental exposures, including pollutants and pathogens, have the potential to disrupt these regulatory mechanisms, resulting in impaired immune development and an elevated susceptibility to IBD (Lepeule et al., 2024). These findings highlight the critical importance of comprehending the influence of early microbial exposures on long-term health outcomes. This is particularly relevant in the context of IBD, where the interactions among genetic, environmental, and epigenetic factors are intricate and multifaceted.

Beyond microbial exposure, early environmental interventions have been recognized as potential strategies for mitigating the risk of IBD. Research indicates that interventions targeting modifications in the early-life environment, such as encouraging breastfeeding and reducing antibiotic usage, may confer protective effects against the onset of IBD (Hufnagel et al., 2022). For instance, extended breastfeeding has been correlated with a reduced incidence of IBD, potentially attributable to the advantageous effects of breast milk on the development of gut microbiota and the immune system (Borowitz, 2022). Furthermore, minimizing exposure to environmental toxins and pollutants during crucial developmental periods is instrumental in influencing health outcomes. Research suggests that prenatal exposure to specific environmental chemicals may result in enduring modifications to immune function and elevate the risk of chronic diseases, such as IBD (Zachariah et al., 2024). Consequently, early environmental interventions possess the capacity to affect immediate health outcomes and may also function as a preventive strategy against the future development of IBD. This underscores the necessity for public health initiatives aimed at enhancing maternal and infant health environments.

The interaction between early life environmental exposures and the onset of IBD represents an expanding area of research, underscoring the importance of both prenatal and early postnatal environments. Elucidating the mechanisms through which these exposures affect immune development and disease susceptibility is essential for the formulation of targeted preventive strategies. Ongoing research should further investigate the intricate relationships among genetic, epigenetic, and environmental factors, as these findings will be critical for shaping clinical practices and public health policies. Such advancements aim to decrease the incidence of IBD and enhance health outcomes for future generations.

## 6 The impact of gut microbiome–DNA methylation interactions on immune regulation

### 6.1 Epigenetic regulation of regulatory T cells and Th17 cells

The differentiation and functional equilibrium between regulatory T cells (Tregs) and T helper 17 (Th17) cells are pivotal for sustaining immune homeostasis and averting autoimmune pathologies. Epigenetic modifications, notably DNA methylation and histone modifications, are instrumental in modulating the expression of key transcription factors that govern the differentiation of these T cell subsets. For example, the transcription factor RORγt, which is crucial for Th17 cell differentiation, is stringently regulated by diverse epigenetic mechanisms, including histone acetylation and methylation. Empirical studies have demonstrated that the presence of specific cytokines, such as IL-6 and TGF-*β*, can alter the epigenetic landscape of naïve CD4 + T cells, thereby promoting their differentiation into either Tregs or Th17 cells, contingent upon the local microenvironment (Chen et al., 2024). Furthermore, the methylation status of genes linked to Tregs function, notably Foxp3, is essential for preserving their suppressive abilities. In the context of inflammatory diseases, a disruption in the Treg/Th17 cell ratio can result in heightened inflammation, underscoring the significance of elucidating the epigenetic regulation of these cells in disease pathogenesis (Palatella et al., 2025; Lopez Krol et al., 2022).

The impact of microbial metabolites on the immune system adds a layer of complexity to the regulation of Tregs and Th17 cells. SCFAs, which are produced by the fermentation of dietary fibers by gut microbiota, exemplify this influence by promoting the differentiation of Tregs while concurrently inhibiting the development of Th17 cells. This modulatory effect is facilitated through epigenetic mechanisms, such as histone modifications, which enhance the expression of genes associated with Treg differentiation (Duddu et al., 2022; Al-Hawary et al., 2023). Conversely, dysbiosis can result in elevated Th17 cell responses and a reduction in Treg populations, thereby contributing to the pathogenesis of conditions such as IBD and other autoimmune disorders (Tang et al., 2022; Huang et al., 2020).

Furthermore, research has elucidated the function of particular epigenetic regulators, notably the histone methyltransferase Setd2, which has been demonstrated to inhibit Th17 cell differentiation while facilitating Treg cell polarization via phospholipid remodeling (Chen et al., 2024). This suggests that targeting epigenetic regulators could provide therapeutic strategies for re-establishing the equilibrium between Tregs and Th17 cells in autoimmune diseases. Furthermore, the interaction between environmental factors, including dietary components and gut microbiota, and the epigenetic regulation of T cell differentiation highlights the complexity of immune responses and the potential for innovative interventions designed to modulate these pathways (Gao et al., 2024; Hu et al., 2022).

### 6.2 Methylation regulation mechanisms of inflammatory factor expression

The methylation status of pivotal inflammatory genes is crucial in modulating their expression levels, thereby impacting the overall inflammatory response. Methylation, an epigenetic modification, can either repress or activate gene expression, contingent upon the context and the specific genes involved. For example, the promoter regions of pro-inflammatory cytokine genes, such as TNF-*α* and IL-6, may undergo methylation alterations that substantially influence their transcriptional activity (Meng et al., 2024). In the context of inflammatory bowel disease (IBD), research has demonstrated that abnormal methylation patterns in cytokine genes are associated with the severity and progression of the disease. Hypermethylation of the TNF-α gene promoter is associated with diminished expression of this cytokine, a key component of the inflammatory response in inflammatory bowel disease (IBD). In contrast, hypomethylation may result in the upregulation of inflammatory cytokines, thereby aggravating the condition. Moreover, environmental factors, such as microbial dysbiosis, have been demonstrated to induce alterations in DNA methylation, which subsequently influence the expression of inflammatory genes, thus playing a role in chronic inflammation and disease pathogenesis (Yan et al., 2023). These findings highlight the critical importance of comprehensively understanding the methylation landscape of inflammatory genes, as such knowledge may offer valuable insights into potential therapeutic targets for modulating inflammatory responses.

Microbial-induced alterations in DNA methylation hold significant relevance within the framework of inflammation. The gut microbiota exerts an influence on the host’s epigenetic architecture, resulting in modifications to the expression of inflammatory mediators. For instance, certain bacterial metabolites have been demonstrated to modulate the activity of DNA methyltransferases—enzymes responsible for the addition of methyl groups to DNA—thereby impacting the expression of genes associated with the inflammatory response (Zhang and Jagannath, 2025). Microbial-induced alterations in methylation can trigger a cascade of inflammatory signaling pathways, thereby promoting a persistent inflammatory state characteristic of diseases such as inflammatory bowel disease (IBD). Furthermore, the interaction between inflammation and methylation is particularly evident in conditions such as sepsis, where inflammatory cytokines can exacerbate methylation changes, establishing a feedback loop that perpetuates inflammation and tissue damage (Wang et al., 2023). Comprehending these mechanisms is essential for the development of targeted interventions capable of modulating the inflammatory response via epigenetic pathways.

### 6.3 Microbial-mediated mechanisms of host immune tolerance

The interaction between gut microbiota and host immune tolerance represents a complex and dynamic process, wherein microbial communities are integral to the regulation of immune responses. Microorganisms exert influence on immune tolerance through diverse mechanisms, notably by modulating epigenetic factors such as DNA methylation, which can subsequently alter gene expression profiles within host immune cells. For example, microbial metabolites generated during the fermentation of dietary fibers have been demonstrated to facilitate the differentiation and function of Tregs (Sharma et al., 2025). Tregs play a crucial role in sustaining immune homeostasis and averting hyperactive immune responses to non-pathogenic antigens, including those from commensal bacteria. The establishment of a diverse and balanced gut microbiota is vital for the development of Tregs. Conversely, dysbiosis can disrupt immune tolerance and is associated with the onset of inflammatory conditions such as IBD (Heidari et al., 2024). Moreover, microbial constituents, including polysaccharides and peptidoglycans, are capable of directly engaging with host immune receptors, thereby initiating signaling pathways that augment immune tolerance. For instance, the detection of microbial antigens by Toll-like receptors (TLRs) on immune cells can stimulate the secretion of anti-inflammatory cytokines, thereby promoting a tolerogenic milieu (Riazi-Rad et al., 2021).

The breakdown of immune tolerance is a pivotal element in the pathogenesis of IBD, characterized by the immune system’s inappropriate responses against the gut microbiota, resulting in chronic inflammation and tissue damage. Studies suggest that the dysregulation of immune tolerance mechanisms is frequently linked to distinct alterations in the composition of gut microbiota, which may further intensify inflammatory responses (Rezende et al., 2023). For example, an elevation in pro-inflammatory bacterial species, including specific strains of *Escherichia coli*, has been associated with enhanced immune activation and the pathogenesis of IBD. Conversely, beneficial microorganisms, such as *Faecalibacterium prausnitzii*, are frequently observed in reduced abundance in individuals with the condition (Aldars-García et al., 2021). This dysbiosis not only perturbs the equilibrium of immune responses but also compromises epithelial barrier function, facilitating enhanced microbial translocation and subsequent immune activation (Alswat, 2024).

Recent evidence indicates that therapeutic strategies focused on restoring microbial equilibrium may improve immune tolerance and alleviate the inflammatory processes associated with IBD. Interventions including dietary modifications, probiotics, and fecal microbiota transplantation (FMT) have demonstrated potential in rebalancing the gut microbiota and fostering a more favorable immune profile (Zuo et al., 2024). For example, FMT has been linked to substantial clinical improvements in patients experiencing recurrent *Clostridium difficile* infections and has been investigated as a potential therapeutic approach for IBD (Heidari et al., 2024). Furthermore, the modulation of gut microbiota via the administration of targeted prebiotics and probiotics can augment the production of SCFAs, thereby facilitating the differentiation and function of Tregs (Sharma et al., 2025).

## 7 Microbial-epigenetic targeted therapy strategies

### 7.1 Application of probiotics, prebiotics, and postbiotics

In recent years, the utilization of probiotics, prebiotics, and postbiotics has attracted considerable scholarly interest due to their potential to restore gut microbiota equilibrium and positively impact host health. Probiotics, defined as live microorganisms, have demonstrated the ability to enhance gut health by modulating the composition of the microbiota, thereby restoring eubiosis in individuals experiencing dysbiosis, a condition frequently linked to IBD (Tie et al., 2023). Prebiotics, conversely, are non-digestible dietary constituents that selectively enhance the proliferation and activity of advantageous gut microbiota, thereby facilitating the production of SCFAs and other metabolites that promote gastrointestinal health and overall well-being (Kim and Mills, 2024). The interaction among these biotic compounds contributes to the restoration of microbial equilibrium and holds significant implications for epigenetic modifications, particularly concerning DNA methylation. This process can subsequently affect gene expression associated with inflammation and immune responses (Zhang et al., 2025). Moreover, the utilization of postbiotics—bioactive compounds resulting from the metabolic processes of probiotics—has gained recognition as an innovative therapeutic approach. These postbiotics, encompassing metabolites such as SCFAs, proteins, and polysaccharides, have demonstrated advantageous effects on the host, including immunomodulatory, anti-inflammatory, and antioxidant properties (Kumar et al., 2024).

In the management of IBD, probiotics, prebiotics, and postbiotics exhibit unique potential as essential elements of therapeutic strategies aimed at microbial epigenetics. Probiotics influence gut microbiota and contribute to the maintenance of intestinal homeostasis. Although the precise anti-inflammatory mechanisms of probiotics in IBD are not yet fully elucidated, research indicates that extracellular vesicles (EVs) derived from probiotics may act as mediators of host immune responses and exert anti-inflammatory effects (Hao et al., 2021). However, probiotics demonstrate beneficial effects during active ulcerative colitis but show limited efficacy during remission and in Crohn’s disease (Liu et al., 2021). Furthermore, safety concerns surround probiotic use, prompting postbiotics as an alternative clinical option (Zhang et al., 2022). Postbiotics offer distinct advantages in stability, safety, and broad applicability, primarily acting through pathogen suppression, enhanced intestinal barrier function, and/or host immune modulation (Zhong et al., 2022). Research indicates that postbiotics exert stronger regulatory effects than probiotics in modulating host gut microbiota and metabolic pathways, particularly in ulcerative colitis management (Zhang et al., 2022). Prebiotics, by mimicking gut microbial signals or modifying the microbiota, offer novel therapeutic options for IBD (Person and Keefer, 2021). Overall, probiotics, prebiotics, and postbiotics each possess distinct characteristics in IBD therapy. Postbiotics, owing to their safety and efficacy, may emerge as the next generation of biological therapeutics (Zhang et al., 2022; Zhong et al., 2022).

### 7.2 The potential of Fecal microbiota transplantation (FMT) in IBD

FMT has gained recognition as a promising therapeutic approach for IBD, primarily due to its capacity to restore the gut microbiota composition and potentially ameliorate the dysbiosis associated with these conditions. The fundamental mechanism of FMT involves the transfer of healthy fecal microbiota from a donor into the recipient’s gastrointestinal tract, thereby re-establishing a balanced microbial community. This restoration is critical, as dysbiosis has been implicated in the pathogenesis of IBD, contributing to chronic inflammation and immune dysregulation (Xiao et al., 2022). Research indicates that FMT can effectively reconfigure the gut microbiome, facilitating the reestablishment of normal methylation patterns frequently disrupted in patients with IBD (Parenteral and Enteral Nutrition Branch of the Chinese Medical Association, Chinese Society for the Promotion of Human Health Science and Technology, Committee on Gut Microecology and Fecal Microbiota Transplantation, Shanghai Preventive Medicine Association, 2025). The restoration of microbial diversity not only enhances gut health but also reestablishes host-microbiota communication, a critical factor in maintaining mucosal immunity and preventing inflammation.

Clinical trials have yielded considerable evidence affirming the efficacy of FMT in mitigating symptoms of IBD and enhancing immune function. For example, a systematic review and meta-analysis indicated that FMT elicited a significant clinical response in patients with ulcerative colitis, with remission rates reaching as high as 37% (Caldeira et al., 2020). Moreover, FMT has been correlated with enhancements in quality of life and a decreased reliance on corticosteroids and other immunosuppressive therapies, which are frequently employed in the management of IBD (Benech and Sokol, 2020). The underlying mechanisms responsible for these clinical benefits are complex and likely involve the modulation of inflammatory pathways, the restoration of the intestinal barrier, and the alteration of microbial metabolites that impact host immune responses (Gray et al., 2024).

Although the results appear promising, the implementation of FMT in the treatment of IBD presents several challenges. Variability in donor selection, preparation protocols, and recipient characteristics can substantially influence the outcomes of FMT procedures (Zhang et al., 2024). Furthermore, apprehensions about the long-term safety of FMT, particularly the risk of adverse events such as infections or the transmission of pathogenic organisms, underscore the importance of rigorous donor screening and meticulous post-transplant monitoring of recipients (Peery et al., 2024). Recent consensus guidelines have sought to standardize the FMT process by providing recommendations on donor selection, stool processing, and administration routes, with the objective of improving the safety and efficacy of this therapeutic approach (Parenteral and Enteral Nutrition Branch of the Chinese Medical Association, Chinese Society for the Promotion of Human Health Science and Technology, Committee on Gut Microecology and Fecal Microbiota Transplantation, Shanghai Preventive Medicine Association, 2025).

### 7.3 Epigenetic drugs and microbiome therapy combined application

The interaction between epigenetic modifications and gut microbiota has become a pivotal focus of research concerning IBD and its treatment. Epigenetic pharmacological agents, notably DNA methyltransferase inhibitors, have been recognized as potential therapeutic candidates capable of reversing abnormal DNA methylation patterns implicated in disease pathogenesis. For example, compounds such as decitabine have demonstrated potential in modulating gene expression by inhibiting DNA methylation, thereby restoring normal cellular functions and potentially mitigating inflammatory responses in IBD (Gupta et al., 2025). The utilization of these pharmacological agents is especially pertinent in the context of IBD, wherein dysregulated gene expression resulting from epigenetic modifications significantly contributes to disease progression. Nonetheless, epigenetic therapies have the potential to extensively alter the genome, thereby causing off-target effects. For instance, inhibitors of DNA methylation may concurrently activate both pro-inflammatory and anti-inflammatory genes (Guo et al., 2025). Consequently, the incorporation of microbiome therapy represents a novel strategy to augment the therapeutic efficacy of epigenetic drugs. The gut microbiota is recognized for its capacity to modulate host epigenetic landscapes via the production of metabolites that influence DNA methylation and histone modifications (Zhang et al., 2025). This interaction between microbiome-derived metabolites and epigenetic pharmaceuticals holds the potential to enhance the efficacy and personalization of treatment strategies for IBD.

Furthermore, the integration of microbiome interventions with epigenetic therapies holds the potential to enhance therapeutic precision. Recent research has demonstrated that FMT can induce substantial alterations in gut microbiome composition, subsequently impacting systemic epigenetic profiles. In individuals with systemic lupus erythematosus, FMT has been observed to elevate global DNA methylation levels, indicating that the restoration of a healthy microbiome may exert extensive effects on epigenetic regulation (Zhang et al., 2023). This phenomenon highlights the potential of microbiome therapy to not only restore microbial equilibrium but also to augment the efficacy of epigenetic drugs by establishing an environment conducive to their action. Moreover, dietary modifications that promote beneficial gut microbiota may serve as adjunctive strategies alongside epigenetic therapies. For example, fiber-rich diets can enhance the production of beneficial metabolites, thereby supporting the therapeutic effects of DNA methyltransferase inhibitors and other epigenetic agents (Dong et al., 2025). Nevertheless, it is important to note that dietary fiber does not uniformly benefit all individuals with IBD. Certain patients experience intolerance to fiber, a condition that may be linked to a diminished presence of fiber-fermenting microorganisms within the gut microbiota (Armstrong et al., 2023; Bonazzi et al., 2024). Consequently, a personalized evaluation of fiber type and dosage is essential. Clinical guidelines recommend augmenting fiber intake during the remission phase of IBD, while advising against its consumption during active disease episodes, as it may exacerbate symptoms (Peters et al., 2022). At present, there are no specific guidelines concerning the types of fiber recommended for various stages of disease (Peters et al., 2022).

In conclusion, the integrated application of epigenetic drugs and microbiome therapy constitutes a promising frontier in the treatment of IBD. By harnessing the synergistic effects of these two therapeutic modalities, it is conceivable to attain more effective and personalized treatment outcomes. Nevertheless, the majority of epigenetic interventions are presently in the experimental phase and necessitate higher-level evidence to substantiate their efficacy (Zhang et al., 2025; Nohesara et al., 2025).

## 8 Limitations and future directions

Despite significant progress in understanding the interaction between gut microbiota and host DNA methylation in IBD, several limitations persist. Current studies often rely on animal models or small human cohorts, limiting the generalizability of findings to diverse populations (Zhang et al., 2025; Dennison et al., 2024). The causal relationship between microbial-induced epigenetic changes and IBD pathogenesis remains unclear, as most evidence is correlative (Chen et al., 2025; Woo and Alenghat, 2022). Additionally, the complexity of microbiota-host interactions, influenced by diet, genetics, and environmental factors, poses challenges in isolating specific mechanistic pathways (Guo et al., 2025). Multi-omics approaches (e.g., metagenomics, methylomics) are promising but require standardization to reconcile discrepancies across studies (Liu et al., 2024). Furthermore, while FMT and dietary interventions show potential in modulating DNA methylation (Stols-Gonçalves et al., 2023), their long-term efficacy and safety in IBD therapy need rigorous validation.

Future research should prioritize large-scale longitudinal studies to delineate temporal dynamics of microbiota-epigenome crosstalk in IBD progression (Joustra et al., 2023; Ventham et al., 2023). Single-cell epigenomic profiling could resolve cell-type-specific methylation patterns, particularly in intestinal epithelial and immune cells (Dennison et al., 2024; Sun et al., 2023). Mechanistic studies leveraging gnotobiotic models or *in vitro* systems are needed to validate microbial metabolites (e.g., SCFAs) as epigenetic regulators (Tian and Chen, 2024). Clinically, integrating microbiome-epigenome biomarkers could refine personalized therapies, such as probiotic or phage-based interventions (Lazarević et al., 2025). Finally, exploring the reversibility of microbial-driven epigenetic dysregulation may unveil novel therapeutic targets for sustained remission (Fazio et al., 2022).

## 9 Conclusion

The complex interaction between gut microbiota and host DNA methylation is pivotal in the pathogenesis of IBD, as it jointly regulates immune responses and the integrity of the intestinal barrier. Environmental factors indirectly influence the host’s epigenetic state by affecting the microbiota and their metabolites, highlighting the need to consider external variables in disease management. Advances in multi-omics technologies offer powerful tools for elucidating this interaction network. By integrating multi-layered data, these technologies facilitate precision diagnosis and personalized treatment of IBD. Although combined interventions targeting both the microbiota and DNA methylation hold significant promise, their clinical translation is challenged by complex mechanisms and individual variability. Moving forward, it is essential to prioritize the integration of multi-omics data, develop personalized treatment plans, and conduct rigorous clinical safety assessments. Through interdisciplinary collaboration and patient-centered strategies, we can ultimately achieve the overarching goals of reducing the disease burden and enhancing the quality of life for patients.

## References

[ref1] Akanyibah F. A. Zhu Y. Wan A. Ocansey D. K. W. Xia Y. Fang A. N. . (2024). Effects of DNA methylation and its application in inflammatory bowel disease (review). Int. J. Mol. Med. 53:55. doi: 10.3892/ijmm.2024.5379, PMID: 38695222 PMC11093555

[ref2] Aldars-García L. Marin A. C. Chaparro M. Gisbert J. P. (2021). The interplay between immune system and microbiota in inflammatory bowel disease: a narrative review. Int. J. Mol. Sci. 22:3076. doi: 10.3390/ijms22063076, PMID: 33802883 PMC8002696

[ref3] Alharthi A. Alhazmi S. Alburae N. Bahieldin A. (2022). The human gut microbiome as a potential factor in autism Spectrum disorder. Int. J. Mol. Sci. 23:1363. doi: 10.3390/ijms23031363, PMID: 35163286 PMC8835713

[ref4] Al-Hawary S. I. S. Kashikova K. Ioffe E. M. Izbasarova A. Hjazi A. Tayyib N. A. . (2023). Pathological role of LncRNAs in immune-related disease via regulation of T regulatory cells. Pathol. Res. Pract. 249:154709. doi: 10.1016/j.prp.2023.154709, PMID: 37586216

[ref5] Alsharairi N. A. (2023). Therapeutic potential of gut microbiota and its metabolite short-chain fatty acids in neonatal necrotizing enterocolitis. Life (Basel, Switzerland) 13:561. doi: 10.3390/life13020561, PMID: 36836917 PMC9959300

[ref6] Alswat A. S. (2024). The influence of the gut microbiota on host health: a focus on the gut-lung axis and therapeutic approaches. Life (Basel, Switzerland) 14:1279. doi: 10.3390/life14101279, PMID: 39459579 PMC11509314

[ref7] Ansari I. Solé-Boldo L. Ridnik M. Gutekunst J. Gilliam O. Korshko M. . (2023). TET2 and TET3 loss disrupts small intestine differentiation and homeostasis. Nat. Commun. 14:4005. doi: 10.1038/s41467-023-39512-3, PMID: 37414790 PMC10326054

[ref8] Armstrong H. K. Bording-Jorgensen M. Santer D. M. Zhang Z. Valcheva R. Rieger A. M. . (2023). Unfermented β-fructan Fibers fuel inflammation in select inflammatory bowel disease patients. Gastroenterology 164, 228–240. doi: 10.1053/j.gastro.2022.09.034, PMID: 36183751

[ref9] Benech N. Sokol H. (2020). Fecal microbiota transplantation in gastrointestinal disorders: time for precision medicine. Genome Med. 12:58. doi: 10.1186/s13073-020-00757-y, PMID: 32605650 PMC7329555

[ref10] Bi Y. Cheng B. Zou B. Liu S. Cui Z. (2025). The current landscape of fecal microbiota transplantation in treating inflammatory bowel disease. Transl. Gastroenterol. Hepatol. 10:55. doi: 10.21037/tgh-24-138, PMID: 40755720 PMC12314702

[ref11] Bonazzi E. Bretin A. Vigué L. Hao F. Patterson A. D. Gewirtz A. T. . (2024). Individualized microbiotas dictate the impact of dietary fiber on colitis sensitivity. Microbiome 12:5. doi: 10.1186/s40168-023-01724-6, PMID: 38178260 PMC10768099

[ref12] Borowitz S. M. (2022). The epidemiology of inflammatory bowel disease: clues to pathogenesis? Front. Pediatr. 10:1103713. doi: 10.3389/fped.2022.1103713, PMID: 36733765 PMC9886670

[ref13] Caldeira L. F. Borba H. H. Tonin F. S. Wiens A. Fernandez-Llimos F. Pontarolo R. (2020). Fecal microbiota transplantation in inflammatory bowel disease patients: a systematic review and meta-analysis. PLoS One 15:e0238910. doi: 10.1371/journal.pone.0238910, PMID: 32946509 PMC7500646

[ref14] Ceballos D. Hernández-Camba A. Ramos L. (2021). Diet and microbiome in the beginning of the sequence of gut inflammation. World J. Clin. Cases 9, 11122–11147. doi: 10.12998/wjcc.v9.i36.11122, PMID: 35071544 PMC8717522

[ref15] Chargo N. J. Kang H. J. Das S. Jin Y. Rockwell C. Cho J. Y. . (2024). Korean red ginseng extract prevents bone loss in an oral model of glucocorticoid induced osteoporosis in mice. Front. Pharmacol. 15:1268134. doi: 10.3389/fphar.2024.1268134, PMID: 38533264 PMC10963623

[ref16] Chen Y. Chen X. Lin S. Huang S. Li L. Hong M. . (2025). Effects of psychological stress on inflammatory bowel disease via affecting the microbiota-gut-brain axis. Chin. Med. J. 138, 664–677. doi: 10.1097/CM9.0000000000003389, PMID: 39965932 PMC11925421

[ref17] Chen Y. Chen K. Zhu H. Qin H. Liu J. Cao X. (2024). Methyltransferase Setd2 prevents T cell-mediated autoimmune diseases via phospholipid remodeling. Proc. Natl. Acad. Sci. USA 121:e2314561121. doi: 10.1073/pnas.2314561121, PMID: 38359295 PMC10895270

[ref18] Chen A. Jiang Z. Cai L. Tang D. (2023). On the road to colorectal cancer development: crosstalk between the gut microbiota, metabolic reprogramming, and epigenetic modifications. Carcinogenesis 44, 631–641. doi: 10.1093/carcin/bgad058, PMID: 37586059

[ref19] Chen N. Miao L. Lin W. Zou D. Huang L. Huang J. . (2021). Integrated DNA methylation and gene expression analysis identified S100A8 and S100A9 in the pathogenesis of obesity. Front. Cardiovasc. Med. 8:631650. doi: 10.3389/fcvm.2021.631650, PMID: 34055926 PMC8163519

[ref20] Cheng B. Rong A. M. Li W. Bi X. Qiu X. (2022). DNMT3a-mediated enterocyte barrier dysfunction contributes to ulcerative colitis via facilitating the interaction of enterocytes and B cells. Mediat. Inflamm. 2022:4862763. doi: 10.1155/2022/4862763, PMID: 35574272 PMC9106515

[ref21] Corridoni D. Chapman T. Antanaviciute A. Satsangi J. Simmons A. (2020). Inflammatory bowel disease through the Lens of single-cell RNA-seq technologies. Inflamm. Bowel Dis. 26, 1658–1668. doi: 10.1093/ibd/izaa089, PMID: 32386055 PMC10686606

[ref22] Dennison T. W. Edgar R. D. Payne F. Nayak K. M. Ross A. D. B. Cenier A. . (2024). Patient-derived organoid biobank identifies epigenetic dysregulation of intestinal epithelial MHC-I as a novel mechanism in severe Crohn's disease. Gut 73, 1464–1477. doi: 10.1136/gutjnl-2024-332043, PMID: 38857990 PMC11347221

[ref23] Dong S. Li X. Huang Q. Li Y. Li J. Zhu X. . (2025). Resistance to immunotherapy in non-small cell lung cancer: unraveling causes, developing effective strategies, and exploring potential breakthroughs. Drug Resist. Updat. 81:101215. doi: 10.1016/j.drup.2025.101215, PMID: 40081220

[ref24] Duddu A. S. Majumdar S. S. Sahoo S. Jhunjhunwala S. Jolly M. K. (2022). Emergent dynamics of a three-node regulatory network explain phenotypic switching and heterogeneity: a case study of Th1/Th2/Th17 cell differentiation. Mol. Biol. Cell 33:ar46. doi: 10.1091/mbc.E21-10-0521, PMID: 35353012 PMC9265159

[ref25] Fazio A. Bordoni D. Kuiper J. W. P. Weber-Stiehl S. Stengel S. T. Arnold P. . (2022). DNA methyltransferase 3A controls intestinal epithelial barrier function and regeneration in the colon. Nat. Commun. 13:6266. doi: 10.1038/s41467-022-33844-2, PMID: 36271073 PMC9587301

[ref26] Fei S. F. Hou C. Jia F. (2024). Effects of salidroside on atherosclerosis: potential contribution of gut microbiota. Front. Pharmacol. 15:1400981. doi: 10.3389/fphar.2024.1400981, PMID: 39092226 PMC11292615

[ref27] Fu Y. Lyu J. Wang S. (2023). The role of intestinal microbes on intestinal barrier function and host immunity from a metabolite perspective. Front. Immunol. 14:1277102. doi: 10.3389/fimmu.2023.1277102, PMID: 37876938 PMC10591221

[ref28] Gao Q. Ren J. Niu D. Guo L. Feng X. (2024). Role of protein post-translational modifications in unexplained recurrent pregnancy loss. J. Central South Univ. Med. Sci. 49, 1495–1502. doi: 10.11817/j.issn.1672-7347.2024.240365 PMC11814392 39931779

[ref29] Gill P. A. Inniss S. Kumagai T. Rahman F. Z. Smith A. M. (2022). The role of diet and gut microbiota in regulating gastrointestinal and inflammatory disease. Front. Immunol. 13:866059. doi: 10.3389/fimmu.2022.866059, PMID: 35450067 PMC9016115

[ref30] Gray S. M. Moss A. D. Herzog J. W. Kashiwagi S. Liu B. Young J. B. . (2024). Mouse adaptation of human inflammatory bowel diseases microbiota enhances colonization efficiency and alters microbiome aggressiveness depending on the recipient colonic inflammatory environment. Microbiome 12:147. doi: 10.1186/s40168-024-01857-2, PMID: 39113097 PMC11304999

[ref31] Grycová A. Joo H. Maier V. Illés P. Vyhlídalová B. Poulíková K. . (2022). Targeting the aryl hydrocarbon receptor with microbial metabolite mimics alleviates experimental colitis in mice. J. Med. Chem. 65, 6859–6868. doi: 10.1021/acs.jmedchem.2c00208, PMID: 35416668

[ref32] Guo X. Li J. Xu J. Zhang L. Huang C. Nie Y. . (2025). Gut microbiota and epigenetic inheritance: implications for the development of IBD. Gut Microbes 17:2490207. doi: 10.1080/19490976.2025.2490207, PMID: 40213833 PMC12931708

[ref33] Gupta M. K. Gouda G. Moazzam-Jazi M. Vadde R. Nagaraju G. P. El-Rayes B. F. (2025). CRISPR/Cas9-directed epigenetic editing in colorectal cancer. Biochim. Biophys. Acta Rev. Cancer 1880:189338. doi: 10.1016/j.bbcan.2025.189338, PMID: 40315964

[ref34] Hao H. Zhang X. Tong L. Liu Q. Liang X. Bu Y. . (2021). Effect of extracellular vesicles derived from *Lactobacillus plantarum* Q7 on gut microbiota and ulcerative colitis in mice. Front. Immunol. 12:777147. doi: 10.3389/fimmu.2021.777147, PMID: 34925349 PMC8674835

[ref35] Heidari M. Maleki Vareki S. Yaghobi R. Karimi M. H. (2024). Microbiota activation and regulation of adaptive immunity. Front. Immunol. 15:1429436. doi: 10.3389/fimmu.2024.1429436, PMID: 39445008 PMC11496076

[ref36] Hong J. Rhee J. K. (2022). Genomic effect of DNA methylation on gene expression in colorectal Cancer. Biology 11:1388. doi: 10.3390/biology11101388, PMID: 36290295 PMC9598958

[ref37] Hu S. Liu H. Tao S. Wang Y. Wang S. Liao Y. . (2022). The environmental pollutant 3-methyl-4-nitrophenol reduces the regulatory T cells in the intestine. Toxicology 482:153356. doi: 10.1016/j.tox.2022.153356, PMID: 36283488

[ref38] Huang Y. Wang H. Ba X. Chen Z. Wang Y. Qin K. . (2020). Decipher manifestations and Treg /Th17 imbalance in multi-staging rheumatoid arthritis and correlation with TSDR/RORC methylation. Mol. Immunol. 127, 1–11. doi: 10.1016/j.molimm.2020.08.002, PMID: 32866740

[ref39] Hufnagel A. Grant I. D. Aiken C. E. M. (2022). Glucose and oxygen in the early intrauterine environment and their role in developmental abnormalities. Semin. Cell Dev. Biol. 131, 25–34. doi: 10.1016/j.semcdb.2022.03.041, PMID: 35410716

[ref40] Iyer N. Corr S. C. (2021). Gut microbial metabolite-mediated regulation of the intestinal barrier in the pathogenesis of inflammatory bowel disease. Nutrients 13:4259. doi: 10.3390/nu13124259, PMID: 34959809 PMC8704337

[ref41] Ji J. Wu X. Li X. Zhu Y. (2023). Effects of microplastics in aquatic environments on inflammatory bowel disease. Environ. Res. 229:115974. doi: 10.1016/j.envres.2023.115974, PMID: 37088319

[ref42] Joustra V. Hageman I. L. Satsangi J. Adams A. Ventham N. T. de Jonge W. J. . (2023). Systematic review and Meta-analysis of peripheral blood DNA methylation studies in inflammatory bowel disease. J. Crohns Colitis 17, 185–198. doi: 10.1093/ecco-jcc/jjac119, PMID: 35998097 PMC10024549

[ref43] Kim Y. T. Mills D. A. (2024). Exploring the gut microbiome: probiotics, prebiotics, synbiotics, and postbiotics as key players in human health and disease improvement. Food Sci. Biotechnol. 33, 2065–2080. doi: 10.1007/s10068-024-01620-1, PMID: 39130661 PMC11315840

[ref44] Kim B. Song A. Son A. Shin Y. (2024). Gut microbiota and epigenetic choreography: implications for human health: a review. Medicine 103:e39051. doi: 10.1097/MD.0000000000039051, PMID: 39029010 PMC11398772

[ref45] Kumar A. Green K. M. Rawat M. (2024). A comprehensive overview of postbiotics with a special focus on discovery techniques and clinical applications. Foods 13:2937. doi: 10.3390/foods13182937, PMID: 39335866 PMC11431132

[ref46] Lazarević S. Đanić M. Pavlović N. (2025). Bidirectional interplay between IBD therapies and the gut microbiota: a pharmacomicrobiomic approach to personalized treatment. BioDrugs 39, 877–897. doi: 10.1007/s40259-025-00739-9, PMID: 41044468

[ref47] Lepeule J. Broséus L. Jedynak P. Masdoumier C. Philippat C. Guilbert A. . (2024). Environmental exposures and epigenome changes within the first 1000 days of life. Med. Sci. 40, 947–954. doi: 10.1051/medsci/2024178, PMID: 39705565

[ref48] Levic D. S. Niedzwiecki D. Kandakatla A. Karlovich N. S. Juneja A. Park J. . (2024). TNF promoter hypomethylation is associated with mucosal inflammation in IBD and anti-TNF response. Gastro Hep Advances 3, 888–898. doi: 10.1016/j.gastha.2024.06.010, PMID: 39286616 PMC11402298

[ref49] Li J. Liang J. Zeng M. Sun K. Luo Y. Zheng H. . (2023). Oxymatrine ameliorates white matter injury by modulating gut microbiota after intracerebral hemorrhage in mice. CNS Neurosci. Ther. 29, 18–30. doi: 10.1111/cns.14066 36550632 PMC10314101

[ref50] Li D. Liu Z. Fan X. Zhao T. Wen D. Huang X. . (2024). Lactic acid Bacteria-gut-microbiota-mediated intervention towards inflammatory bowel disease. Microorganisms 12:1864. doi: 10.3390/microorganisms12091864, PMID: 39338538 PMC11433943

[ref51] Liu Y. Huang Y. He Q. Dou Z. Zeng M. Wang X. . (2023). From heart to gut: exploring the gut microbiome in congenital heart disease. iMeta 2:e144. doi: 10.1002/imt2.144, PMID: 38868221 PMC10989834

[ref52] Liu A. Wang B. Wang M. Tang R. Xu W. Xiao W. (2025). L-theanine alleviates ulcerative colitis by repairing the intestinal barrier through regulating the gut microbiota and associated short-chain fatty acids. Food Chem. Toxicol. 202:115497. doi: 10.1016/j.fct.2025.115497, PMID: 40311999

[ref53] Liu Y. Yin F. Huang L. Teng H. Shen T. Qin H. (2021). Long-term and continuous administration of *Bacillus subtilis* during remission effectively maintains the remission of inflammatory bowel disease by protecting intestinal integrity, regulating epithelial proliferation, and reshaping microbial structure and function. Food Funct. 12, 2201–2210. doi: 10.1039/d0fo02786c, PMID: 33595001

[ref54] Liu Z. Zhang Q. Zhang H. Yi Z. Ma H. Wang X. . (2024). Colorectal cancer microbiome programs DNA methylation of host cells by affecting methyl donor metabolism. Genome Med. 16:77. doi: 10.1186/s13073-024-01344-1, PMID: 38840170 PMC11151592

[ref55] Liu Y. Zhou Y. Zhang H. Zhao K. Yang D. (2025). Gut-lung Axis mediates asthma pathogenesis: roles of dietary patterns and their impact on the gut microbiota. Exp. Mol. Pathol. 142:104964. doi: 10.1016/j.yexmp.2025.104964, PMID: 40194490

[ref56] Lopez Krol A. Nehring H. P. Krause F. F. Wempe A. Raifer H. Nist A. . (2022). Lactate induces metabolic and epigenetic reprogramming of pro-inflammatory Th17 cells. EMBO Rep. 23:e54685. doi: 10.15252/embr.202254685, PMID: 36215678 PMC9724659

[ref57] Meng W. Fenton C. G. Johnsen K. M. Taman H. Florholmen J. Paulssen R. H. (2024). DNA methylation fine-tunes pro-and anti-inflammatory signalling pathways in inactive ulcerative colitis tissue biopsies. Sci. Rep. 14:6789. doi: 10.1038/s41598-024-57440-0, PMID: 38514698 PMC10957912

[ref58] Mostafavi Abdolmaleky H. Zhou J. R. (2024). Gut microbiota dysbiosis, oxidative stress, inflammation, and epigenetic alterations in metabolic diseases. Antioxidants (Basel, Switzerland) 13:985. doi: 10.3390/antiox13080985, PMID: 39199231 PMC11351922

[ref59] Mu C. Zhao Q. Zhao Q. Yang L. Pang X. Liu T. . (2023). Multi-omics in Crohn's disease: new insights from inside. Comput. Struct. Biotechnol. J. 21, 3054–3072. doi: 10.1016/j.csbj.2023.05.010, PMID: 37273853 PMC10238466

[ref60] Narabayashi H. Koma C. Nakata K. Ikegami M. Nakanishi Y. Ogihara J. . (2022). Gut microbiota-dependent adaptor molecule recruits DNA methyltransferase to the TLR4 gene in colonic epithelial cells to suppress inflammatory reactions. Front. Mol. Biosci. 9:1005136. doi: 10.3389/fmolb.2022.1005136, PMID: 36339704 PMC9634067

[ref61] Niekamp P. Kim C. H. (2023). Microbial metabolite dysbiosis and colorectal Cancer. Gut Liver 17, 190–203. doi: 10.5009/gnl220260, PMID: 36632785 PMC10018301

[ref62] Noble A. J. Nowak J. K. Adams A. T. Uhlig H. H. Satsangi J. (2023). Defining interactions between the genome, epigenome, and the environment in inflammatory bowel disease: Progress and prospects. Gastroenterology 165, 44–60.e2. doi: 10.1053/j.gastro.2023.03.238, PMID: 37062395

[ref63] Nohesara S. Mostafavi Abdolmaleky H. Pettinato G. Pirani A. Thiagalingam S. Zhou J. R. (2025). IUPHAR review: eating disorders, gut microbiota dysbiosis and epigenetic aberrations. Pharmacol. Res. 213:107653. doi: 10.1016/j.phrs.2025.107653, PMID: 39970995

[ref64] Otsuki Y. Ouchi K. Takahashi S. Sasaki K. Sakamoto Y. Okita A. . (2022). Altered gene expression due to aberrant DNA methylation correlates with responsiveness to anti-EGFR antibody treatment. Cancer Sci. 113, 3221–3233. doi: 10.1111/cas.15367, PMID: 35403373 PMC9459254

[ref65] Palatella M. Kruse F. Glage S. Bleich A. Greweling-Pils M. Huehn J. (2025). Acsbg1 regulates differentiation and inflammatory properties of CD4+ T cells. Eur. J. Microbiol. Immunol. 15, 21–31. doi: 10.1556/1886.2025.00003, PMID: 39937199 PMC11925188

[ref66] Paraskevaidis I. Tsougos E. Kourek C. (2025). The microbiome connection: a common pathway linking Cancer and heart failure. Biomedicine 13:1297. doi: 10.3390/biomedicines13061297, PMID: 40564016 PMC12189070

[ref67] Parenteral and Enteral Nutrition Branch of the Chinese Medical Association, Chinese Society for the Promotion of Human Health Science and Technology, Committee on Gut Microecology and Fecal Microbiota Transplantation, Shanghai Preventive Medicine Association (2025). Consensus of Chinese experts on gut microbiota and fecal microbiota transplantation in inflammatory bowel disease (2025 edition). Chin. J. Gastrointest. Surg. 28, 225–235. doi: 10.3760/cma.j.cn441530-20241224-00422 40123393

[ref68] Peery A. F. Kelly C. R. Kao D. Vaughn B. P. Lebwohl B. Singh S. . (2024). AGA clinical practice guideline on Fecal microbiota-based therapies for select gastrointestinal diseases. Gastroenterology 166, 409–434. doi: 10.1053/j.gastro.2024.01.008, PMID: 38395525

[ref69] Pei T. Li W. Zhou Z. Zhang Q. Yu G. Yin S. . (2025). The relationship between tryptophan metabolism and gut microbiota: interaction mechanism and potential effects in infection treatment. Microbiol. Res. 298:128211. doi: 10.1016/j.micres.2025.128211, PMID: 40393170

[ref70] Person H. Keefer L. (2021). Psychological comorbidity in gastrointestinal diseases: update on the brain-gut-microbiome axis. Prog. Neuro-Psychopharmacol. Biol. Psychiatry 107:110209. doi: 10.1016/j.pnpbp.2020.110209, PMID: 33326819 PMC8382262

[ref71] Peters V. Dijkstra G. Campmans-Kuijpers M. J. E. (2022). Are all dietary fibers equal for patients with inflammatory bowel disease? A systematic review of randomized controlled trials. Nutr. Rev. 80, 1179–1193. doi: 10.1093/nutrit/nuab062, PMID: 34486663 PMC8990763

[ref72] Premadasa L. S. McDew-White M. Romero L. Gondo B. Drawec J. A. Ling B. . (2025). Epigenetic modulation of the NLRP6 inflammasome sensor as a therapeutic modality to reduce necroptosis-driven gastrointestinal mucosal dysfunction in HIV/SIV infection. Cell Commun. Signal 23:199. doi: 10.1186/s12964-025-02193-0, PMID: 40281523 PMC12023470

[ref73] Rauluseviciute I. Drabløs F. Rye M. B. (2020). DNA hypermethylation associated with upregulated gene expression in prostate cancer demonstrates the diversity of epigenetic regulation. BMC Med. Genet. 13:6. doi: 10.1186/s12920-020-0657-6, PMID: 31914996 PMC6950795

[ref74] Reva K. Laranjinha J. Rocha B. S. (2023). Epigenetic modifications induced by the gut microbiota may result from what we eat: should we talk about precision diet in health and disease? Meta 13:375. doi: 10.3390/metabo13030375, PMID: 36984815 PMC10051796

[ref75] Rezende R. M. Cox L. M. Moreira T. G. Liu S. Boulenouar S. Dhang F. . (2023). Gamma-delta T cells modulate the microbiota and fecal micro-RNAs to maintain mucosal tolerance. Microbiome 11:32. doi: 10.1186/s40168-023-01478-1, PMID: 36814316 PMC9948450

[ref76] Riazi-Rad F. Behrouzi A. Mazaheri H. Katebi A. Ajdary S. (2021). Impact of gut microbiota on immune system. Acta Microbiol. Immunol. Hung. 68, 135–144. doi: 10.1556/030.2021.01532, PMID: 34375301

[ref77] Santonocito R. Paladino L. Vitale A. M. D'Amico G. Zummo F. P. Pirrotta P. . (2024). Nanovesicular mediation of the gut-brain Axis by probiotics: insights into irritable bowel syndrome. Biology 13:296. doi: 10.3390/biology13050296, PMID: 38785778 PMC11117693

[ref78] Sharma A. Sharma G. Im S. H. (2025). Gut microbiota in regulatory T cell generation and function: mechanisms and health implications. Gut Microbes 17:2516702. doi: 10.1080/19490976.2025.2516702, PMID: 40517372 PMC12169050

[ref79] Shi L. Feng Y. Wang J. Xiao R. Wang L. Tian P. . (2024). Innovative mechanisms of micro- and nanoplastic-induced brain injury: emphasis on the microbiota-gut-brain axis. Life Sci. 357:123107. doi: 10.1016/j.lfs.2024.123107, PMID: 39369844

[ref80] Shoham S. Pintel N. Avni D. (2025). Oxidative stress, gut bacteria, and microalgae: a holistic approach to manage inflammatory bowel diseases. Antioxidants (Basel, Switzerland) 14:697. doi: 10.3390/antiox14060697, PMID: 40563329 PMC12189200

[ref81] Stols-Gonçalves D. Mak A. L. Madsen M. S. van der Vossen E. W. J. Bruinstroop E. Henneman P. . (2023). Faecal microbiota transplantation affects liver DNA methylation in non-alcoholic fatty liver disease: a multi-omics approach. Gut Microbes 15:2223330. doi: 10.1080/19490976.2023.2223330, PMID: 37317027 PMC10269428

[ref82] Su Y. H. Huang W. C. Huang T. H. Huang Y. J. Sue Y. K. Huynh T. T. . (2016). Folate deficient tumor microenvironment promotes epithelial-to-mesenchymal transition and cancer stem-like phenotypes. Oncotarget 7, 33246–33256. doi: 10.18632/oncotarget.8910, PMID: 27119349 PMC5078091

[ref83] Sultan S. El-Mowafy M. Elgaml A. Ahmed T. A. E. Hassan H. Mottawea W. (2021). Metabolic influences of gut microbiota dysbiosis on inflammatory bowel disease. Front. Physiol. 12:715506. doi: 10.3389/fphys.2021.715506, PMID: 34646151 PMC8502967

[ref84] Sun Z. Braga-Neto M. B. Xiong Y. Bhagwate A. V. Gibbons H. R. Sagstetter M. R. . (2023). Hypomethylation and overexpression of Th17-associated genes is a Hallmark of intestinal CD4+ lymphocytes in Crohn's disease. J. Crohns Colitis 17, 1847–1857. doi: 10.1093/ecco-jcc/jjad093, PMID: 37280154 PMC10673812

[ref85] Takahashi K. Morita N. Tamano R. Gao P. Iida N. Andoh A. . (2024). Mouse IgA modulates human gut microbiota with inflammatory bowel disease patients. J. Gastroenterol. 59, 812–824. doi: 10.1007/s00535-024-02121-y, PMID: 38874761 PMC11339086

[ref86] Tang F. Zhou Z. Huang K. Deng W. Lin J. Chen R. . (2022). MicroRNAs in the regulation of Th17/Treg homeostasis and their potential role in uveitis. Front. Genet. 13:848985. doi: 10.3389/fgene.2022.848985, PMID: 36186459 PMC9515448

[ref87] Tian S. Chen M. (2024). Global research progress of gut microbiota and epigenetics: bibliometrics and visualized analysis. Front. Immunol. 15:1412640. doi: 10.3389/fimmu.2024.1412640, PMID: 38803501 PMC11128553

[ref88] Tie Y. Huang Y. Chen R. Li L. Chen M. Zhang S. (2023). Current insights on the roles of gut microbiota in inflammatory bowel disease-associated extra-intestinal manifestations: pathophysiology and therapeutic targets. Gut Microbes 15:2265028. doi: 10.1080/19490976.2023.2265028, PMID: 37822139 PMC10572083

[ref89] Ventham N. T. Kennedy N. A. Kalla R. Adams A. T. Noble A. Ennis H. . (2023). Genome-wide methylation profiling in 229 patients with Crohn's disease requiring intestinal resection: epigenetic analysis of the trial of prevention of post-operative Crohn's disease (TOPPIC). Cell. Mol. Gastroenterol. Hepatol. 16, 431–450. doi: 10.1016/j.jcmgh.2023.06.001, PMID: 37331566 PMC10372903

[ref90] Vieujean S. Caron B. Haghnejad V. Jouzeau J. Y. Netter P. Heba A. C. . (2022). Impact of the exposome on the epigenome in inflammatory bowel disease patients and animal models. Int. J. Mol. Sci. 23:7611. doi: 10.3390/ijms23147611, PMID: 35886959 PMC9321337

[ref91] Vural S. Palmisano A. Reinhold W. C. Pommier Y. Teicher B. A. Krushkal J. (2021). Association of expression of epigenetic molecular factors with DNA methylation and sensitivity to chemotherapeutic agents in cancer cell lines. Clin. Epigenetics 13:49. doi: 10.1186/s13148-021-01026-4, PMID: 33676569 PMC7936435

[ref92] Wang X. Ding Y. Li R. Zhang R. Ge X. Gao R. . (2023). N(6)-methyladenosine of Spi2a attenuates inflammation and sepsis-associated myocardial dysfunction in mice. Nat. Commun. 14:1185. doi: 10.1038/s41467-023-36865-7, PMID: 36864027 PMC9979126

[ref93] Wang X. Geng S. (2023). Diet-gut microbial interactions influence cancer immunotherapy. Front. Oncol. 13:1138362. doi: 10.3389/fonc.2023.1138362, PMID: 37035188 PMC10081683

[ref94] Wang W. Gu W. Schweitzer R. Koren O. Khatib S. Tseng G. . (2025). In utero human intestine contains maternally derived bacterial metabolites. Microbiome 13:116. doi: 10.1186/s40168-025-02110-0, PMID: 40329366 PMC12054239

[ref95] Wang Z. Wang S. Liu S. Wang Z. Li F. Bu Q. . (2025). Polystyrene microplastics induce potential toxicity through the gut-mammary axis. NPJ Sci. Food 9:139. doi: 10.1038/s41538-025-00517-5, PMID: 40659623 PMC12259839

[ref96] Wang Z. Zhou L. Zhong X. Jiang Y. Zhang Z. Li W. (2024). Liquid-liquid separation in gut immunity. Front. Immunol. 15:1505123. doi: 10.3389/fimmu.2024.1505123, PMID: 39720729 PMC11666445

[ref97] Woo V. Alenghat T. (2022). Epigenetic regulation by gut microbiota. Gut Microbes 14:2022407. doi: 10.1080/19490976.2021.2022407, PMID: 35000562 PMC8744890

[ref98] Wu H. Mu C. Xu L. Yu K. Shen L. Zhu W. (2024). Host-microbiota interaction in intestinal stem cell homeostasis. Gut Microbes 16:2353399. doi: 10.1080/19490976.2024.2353399, PMID: 38757687 PMC11110705

[ref99] Xiao Y. Zhong X. S. Liu X. Li Q. (2022). Therapeutic evaluation of Fecal microbiota transplantation in an interleukin 10-deficient mouse model. J. Visual. Exp. 182:10.3791/63350. doi: 10.3791/63350, PMID: 35467645 PMC11915499

[ref100] Xu J. Xu H. M. Yang M. F. Liang Y. J. Peng Q. Z. Zhang Y. . (2022). New insights into the epigenetic regulation of inflammatory bowel disease. Front. Pharmacol. 13:813659. doi: 10.3389/fphar.2022.813659, PMID: 35173618 PMC8841592

[ref101] Yan L. Li W. Chen F. Wang J. Chen J. Chen Y. . (2023). Inflammation as a mediator of microbiome dysbiosis-associated DNA methylation changes in gastric premalignant lesions. Phenomics (Cham, Switzerland). 3, 496–501. doi: 10.1007/s43657-023-00118-w, PMID: 37881317 PMC10593640

[ref102] Yang C. Du Y. Ren D. Yang X. Zhao Y. (2021). Gut microbiota-dependent catabolites of tryptophan play a predominant role in the protective effects of turmeric polysaccharides against DSS-induced ulcerative colitis. Food Funct. 12, 9793–9807. doi: 10.1039/D1FO01468D, PMID: 34664583

[ref103] Yang Q. Wang B. Zheng Q. Li H. Meng X. Zhou F. . (2023). A review of gut microbiota-derived metabolites in tumor progression and cancer therapy. Advanc. Sci. (Weinheim, Baden-Wurttemberg, Germany) 10:e2207366. doi: 10.1002/advs.202207366, PMID: 36951547 PMC10214247

[ref104] Yin T. Zhang X. Xiong Y. Li B. Guo D. Sha Z. . (2024). Exploring gut microbial metabolites as key players in inhibition of cancer progression: mechanisms and therapeutic implications. Microbiol. Res. 288:127871. doi: 10.1016/j.micres.2024.127871, PMID: 39137590

[ref105] Zachariah J. P. Jone P. N. Agbaje A. O. Ryan H. H. Trasande L. Perng W. . (2024). Environmental exposures and Pediatric cardiology: a scientific statement from the American Heart Association. Circulation 149, e1165–e1175. doi: 10.1161/CIR.0000000000001234, PMID: 38618723 PMC12312003

[ref106] Zhang J. Gan H. Duan X. Li G. (2024). Targeting the intestinal microbiota: a novel direction in the treatment of inflammatory bowel disease. Biomedicine 12:4799. doi: 10.3390/biomedicines12102340, PMID: 39457652 PMC11504502

[ref107] Zhang K. Jagannath C. (2025). Crosstalk between metabolism and epigenetics during macrophage polarization. Epigenetics Chromatin 18:16. doi: 10.1186/s13072-025-00575-9, PMID: 40156046 PMC11954343

[ref108] Zhang Q. Liu Y. Li Y. Bai G. Pang J. Wu M. . (2025). Implications of gut microbiota-mediated epigenetic modifications in intestinal diseases. Gut Microbes 17:2508426. doi: 10.1080/19490976.2025.2508426, PMID: 40464639 PMC12143691

[ref109] Zhang T. Zhang W. Feng C. Kwok L. Y. He Q. Sun Z. (2022). Stronger gut microbiome modulatory effects by postbiotics than probiotics in a mouse colitis model. NPJ Sci. Food 6:53. doi: 10.1038/s41538-022-00169-9, PMID: 36379940 PMC9666507

[ref110] Zhang B. Zhou W. Liu Q. Huang C. Hu Z. Zheng M. . (2023). Effects of fecal microbiota transplant on DNA methylation in patients with systemic lupus erythematosus. J. Autoimmun. 141:103047. doi: 10.1016/j.jaut.2023.103047, PMID: 37179169

[ref111] Zhao H. Wang D. Zhang Z. Xian J. Bai X. (2022). Effect of gut microbiota-derived metabolites on immune checkpoint inhibitor therapy: enemy or friend? Molecules (Basel, Switzerland) 27:1345. doi: 10.3390/molecules27154799, PMID: 35956752 PMC9369921

[ref112] Zheng Y. Qin C. Wen M. Zhang L. Wang W. (2024). The effects of food nutrients and bioactive compounds on the gut microbiota: a comprehensive review. Foods 13. doi: 10.3390/foods13091345, PMID: 38731716 PMC11083588

[ref113] Zhong Y. Wang S. Di H. Deng Z. Liu J. Wang H. (2022). Gut health benefit and application of postbiotics in animal production. J. Anim. Sci. Biotechnol. 13:38. doi: 10.1186/s40104-022-00688-1, PMID: 35392985 PMC8991504

[ref114] Zhou Y. Zhang D. Cheng H. Wu J. Liu J. Feng W. . (2024). Repairing gut barrier by traditional Chinese medicine: roles of gut microbiota. Front. Cell. Infect. Microbiol. 14:1389925. doi: 10.3389/fcimb.2024.1389925, PMID: 39027133 PMC11254640

[ref115] Zudeh G. Selvestrel D. Bramuzzo M. Cecchin E. D'Andrea M. Stankovic B. . (2025). NLRP3 promoter methylation as a predictive biomarker for glucocorticoid response in patients with inflammatory bowel disease. Biomed. Pharmacother. 183:117824. doi: 10.1016/j.biopha.2025.117824, PMID: 39826354

[ref116] Zuo S. Huang Y. Zou J. (2024). The role of the gut microbiome in modulating immunotherapy efficacy in colorectal cancer. IUBMB Life 76, 1050–1057. doi: 10.1002/iub.2908, PMID: 39135306

